# Measles disease outbreaks are surging again globally in 2026

**DOI:** 10.1080/21505594.2026.2713277

**Published:** 2026-08-10

**Authors:** Hinh Ly

**Affiliations:** Department of Veterinary & Biomedical Sciences, College of Veterinary Medicine, University of Minnesota, Twin Cities, MN, USA

**Keywords:** Respiratory virus, measles virus (MeV), measles-mumps-rubella (MMR) vaccine

According to the World Health Organization (WHO) [1], the number of global measles cases has steadily increased since the COVID-19 pandemic (Table 1). In the United States (US) and other developed countries in Europe and elsewhere, the measles vaccination hesitancy has had a profound impact on the number of children infected with the measles virus (MeV). In the US alone, according to the US Centers for Disease Control and Prevention (CDC) [2], as of 23 July 2026, there are 2,318 confirmed measles cases reported by 44 of 50 US states and the District of Columbia (with a single reported case) (Figure 1), 93% of which (2,153 of 2,318 confirmed cases) were associated with 35 new outbreaks reported in 2026 , with only a relatively small number of the cases (16) reported among international visitors to the US. South Carolina has the most reported measles cases, currently standing at 670, which is followed by Utah with 522 cases. Texas reported the third most cases (188) in 2026. Virginia has seen a recent uptick of 22 new measles cases in early July among the 176 confirmed cases so far in 2026. Florida reported 141 confirmed cases, whereas Pennsylvania reported 134 total cases, and Iowa reported its first measles case of the year in an international traveler (Figure 1). Other states, such as Alaska, Idaho, Montana, Wyoming, Nebraska, Kansas, Oklahoma, Iowa, Missouri, Wisconsin, Illinois, Kentucky, Tennessee, Alabama, Georgia, and several states in the upper Northeast (except for New Hampshire with zero reported cases) reported less than 10 cases each, whereas those in the West coast (Washington, Oregon, California), upper mid-West (North and South Dakotas, Minnesota, Michigan, Ohio), West (Colorado and New Mexico), and South (North Carolina) listed 50–100 cases each, and Maryland in the Northeast reported 11 cases in 2026 (Figure 1). Besides New Hampshire, other states, such as Nevada, Arkansas, Mississippi, Indiana, and West Virginia, have reported zero measles cases thus far in 2026. Compared to 3 fatal cases reported in the US in 2025, there is thus far no fatality associated with MeV infections this year. It is also worth noting that the total number of reported US measles cases in 2026 (2,318) has already surpassed 2,289 cases reported in the previous year (Table 2), which will likely result in the US losing its measles elimination status it received from the WHO in 2000. This determination will be made by the Pan American Health Organization Regional Verification Commission (PAHO VRC), which is an independent body of experts designated by the PAHO (paho.org) to monitor, evaluate, and formally certify the elimination of vaccine-preventable diseases across the Americas.

**Figure 1.**
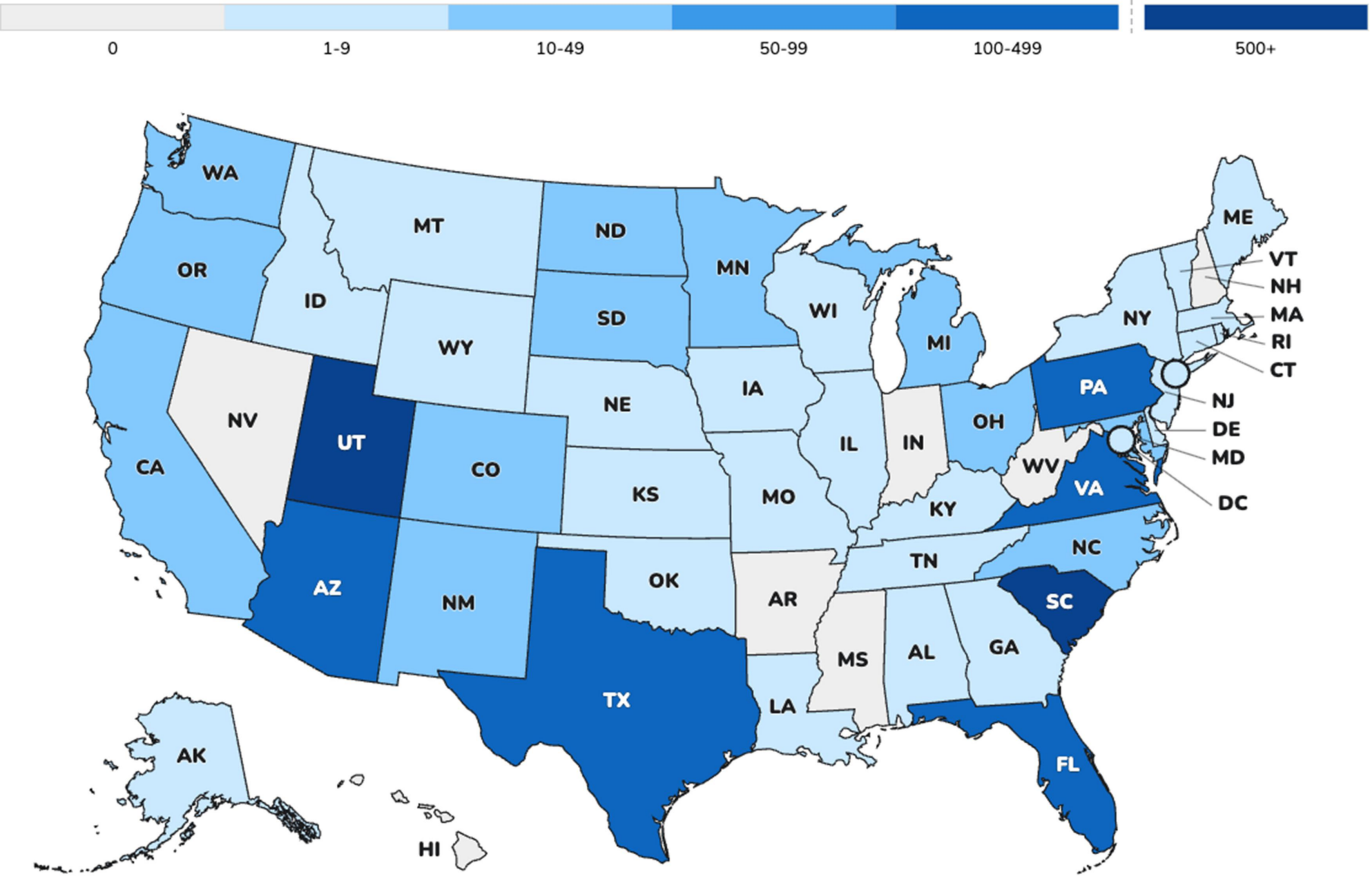
US states and jurisdictions with reported and confirmed cases of measles virus (MeV) infections as of July 23rd, 2026. Figure and data derived and adapted from the US Centers for Disease Control and Prevention (US CDC) [2].

**Table 1.** Reported global cases and incidence of measles are collected annually through the WHO/UNICEF joint reporting form on immunization (JRF). Global aggregate data are released annually in July and updated thereafter as country data are received. Available global measles data from 2020 to 2025 as reported by the WHO [1] are shown.

	Disease	2025	2024	2023	2022	2021	2020
Global	Measles	411,913	476,412	669,083	206,775	123,152	159,240

**Table 2.** Total numbers of measles cases, vaccination status, and hospitalizations in the US in 2025 and 2026. Data derived and adapted from the US Centers for Disease Control and Prevention (US CDC) [2].

	2026 To date	2025 Full year
**Total Cases**	2,318	2,289
**Age**		
Under 5 years	456 (20%)	584 (26%)
5–19 years	1,141 (49%)	1,017 (44%)
20+ years	709 (31%)	675 (29%)
Age unknown	12 (1%)	13 (1%)
**Vaccination Status**		
Unvaccinated or Unknown	93%	93%
One MMR dose	3%	3%
Two MMR doses	4%	4%
U.S. Hospitalizations		
	**2026**	**2025**
**Total Hospitalized**	7% (151 of 2,318 cases)	11% (243 of 2,289 cases)
Percent of Age Group Hospitalized		
Under 5 years	10% (45 of 456)	18% (106 of 584)
5–19 years	3% (38 of 1,141)	6% (58 of 1,017)
20+ years	10% (68 of 709)	12% (79 of 675)
Age unknown	0% (0 of 12)	0% (0 of 13)

The United Kingdom, for example, received its measles elimination status in 2017 due mainly to its relatively high vaccination rates, but lost its status in 2024 following a sharp rise in the number of cases and a significant drop in the 95% vaccination threshold needed in children under five years old to achieve herd immunity. Like in the US, where a combined 70% of the measles cases in 2025–2026 were among children and young adults 19 years old or younger, a significant of whom (13–24%) needed hospitalization (Table 2), about 61% (572 out of 933 cases) reported in England in the early part of 2026 were among children 10 years old or younger [3]. It is noteworthy that 33% of the cases (305 out of 933 cases) were reported in those 15 years old or older living in London and the North West, which was similar to 29–31% of 20 years or older Americans being infected with the MeV in 2025–2026, 10–12% of whom needed medical attention (Table 1). A majority of the total cases (51%, 479 out of 933) in the UK have been reported in London [3], 16% (150 out of 933) in the West Midlands, 12% (111 out of 933) in Enfield, 10% (93 out of 933) in the North West, 9% (81 out of 933) in Birmingham, and 7% (63 out of 933) in Hertfordshire. However, all regions in the UK have reported at least one confirmed case with symptom onset since January 2026. It is also noteworthy that England experienced the highest number of annual measles cases in more than a decade in 2024, when there were 2,911 confirmed cases, whereas there were 22 and 4 confirmed cases reported in Northern Ireland in 2024 and 2025, respectively, and 20 confirmed cases reported in 2024 and only two reported in 2025 in Wales.

Elsewhere in the world, countries such as Bangladesh, India, Yemen, Mexico, Pakistan, Cameroon, Kazakhstan, Guatemala, Angola, and Sudan, have seen the number of measles outbreaks significantly increase in 2025–2026. Bangladesh, for example, which has made significant progress in recent years toward eliminating measles with a campaign to vaccinate over 90% of its children annually, has seen such efforts being undone this year due to factors likely unrelated to vaccine hesitancy, such as overcrowding and a lack of and/or delay in measles-rubella vaccine and vaccination. According to WHO surveillance data [4], Bangladesh recorded 42,174 measles cases from December 2025 to May 2026, followed by India with 26,607 cases, Yemen with 14,521 cases, Mexico with 12,495 cases, Pakistan with 11,037 cases, and other countries (Cameroon, Kazakhstan, Guatemala, Angola, and Sudan) reported less than 10,000 cases each (Table 3). As of late July 2026, the Directorate General of Health Services (DGHS) and humanitarian agencies in Bangladesh have tracked more than 120,000 suspected cases with 14,800 laboratory-confirmed MeV infections and nearly 800 (mostly children) deaths. The United Nations Children’s Fund (UNICEF), a global agency established in 1946 and operating in over 190 countries and territories to provide humanitarian and development aid to children, has said that the true measles case numbers are likely much higher, which has overwhelmed the country’s healthcare infrastructure and disease surveillance and tracking efforts. Together with UNICEF, the Bangladeshi public health agency has ramped up its efforts to vaccinate over 18.4 million children since April 2026, hoping to moderate the rates of infection and death caused by the MeV infection in hard-hit regions of the country.

**Table 3.** Top 10 countries with measles outbreaks in 2026. Data are based on provisional monthly surveillance data reported to the world Health Organization (WHO) as of July 2026. The data shown covered the period between December 2025 and May 2026 and were derived from the US CDC website [4].

Country	Number of Cases
Bangladesh	42,174
India	26,607
Yemen	14,521
Mexico	12,495
Pakistan	11,037
Cameroon	9,653
Kazakhstan	7,730
Guatemala	6,832
Angola	6,815
Sudan	5,766

It is worth emphasizing that measles vaccination remains the most effective measure against this childhood disease to prevent viral transmission in young and immunologically naïve children, but that incomplete vaccination coverage can lead to serious and fatal consequences. In the US, 93% of children infected with MeV in 2025–2026 were either unvaccinated or with unknown vaccination status. Three to four percent of the MeV-infected children had either 1 or 2 doses of the measles-mumps-rubella (MMR) vaccine (Table 2). Severe and deadly measles disease usually affects malnourished young children who are immunocompromised [5]. Vaccination can prevent MeV infection and supportive care can lessen the severity of the disease, but if it is left untreated, serious complications due to MeV infection can lead to blindness, encephalitis, severe diarrhea, dehydration, and secondary respiratory infections, such as bacterial and viral pneumonias. Two doses of the MMR vaccine have been recommended in the US to ensure full and adequate immunity and protection. Measles vaccines, such as the MMR vaccine used in the US, have proven to be safe and effective and have been delivered at least once to about 85% of children worldwide as of 2017, which has led to 80% reduction in childhood deaths due to MeV infections between 2000 and 2017. In other hard-hit regions in Africa, such as Cameroon, Angola, and Sudan, where measles cases and deaths are among the highest in numbers in the world (Table 3), full measles immunization coverage has dropped to 41–45% from 69% of the first dose in 2021–2022. Similarly, a significant decrease in measles vaccine coverage in East, Southeast, and South Asian countries, such as Bangladesh, in recent years (e.g., at 85% for the second dose from 92% for the first dose in 2022) is also equally worrisome.

In this arguably post-COVID-19 pandemic era, it is important to reestablish global efforts to engage and maintain measles vaccination programs toward the common goal of MeV elimination [6]. Measles vaccination programs combined with a robust worldwide surveillance and reporting system should be implemented to mitigate the measles immunity gap. A sustained level of investment and support from local and global communities, as well as from both public and private sectors, is needed to encourage sound public health policies and community engagement to sustain progress toward achieving the global measles elimination goal set forth by the WHO [6].

## Data Availability

No primary data is included in this article.
